# Phaeohyphomycosis in Kidney Transplant Recipients: A Case Series

**DOI:** 10.7759/cureus.82124

**Published:** 2025-04-12

**Authors:** Nishantan Thamotharan, Vengatachalapathy P, Arthipriya Kumaravel, Murugan Sundaram, Adikrishnan Swaminathan

**Affiliations:** 1 Dermatology, Sri Ramachandra Institute of Higher Education and Research, Chennai, IND; 2 Dermatology, Venereology, and Leprosy, Government Medical College and Hospital, Thiruvallur, Chennai, IND

**Keywords:** multiple cysts, phaeohyphomycosis, phialophora verrucosa, renal transplant recipient, verrucous plaque

## Abstract

Phaeohyphomycosis is a rare subcutaneous fungal infection caused by dematiaceous fungi, commonly presenting as cystic swellings or subcutaneous abscesses. These infections predominantly affect immunocompromised individuals, including transplant recipients, due to impaired cell-mediated immunity, which plays a crucial role in fungal defense. We report three cases of post-renal transplant phaeohyphomycosis, presenting within six to 12 months post transplant. The diagnosis was based on clinical features and potassium hydroxide (KOH) mount findings showing pigmented septate hyphae. Histopathological examination of skin biopsy with Gomori methenamine silver stain confirmed pigmented hyphae, and culture identified *Phialophora verrucosa*. Treatment included surgical excision of lesions combined with oral itraconazole and voriconazole.

## Introduction

Phaeohyphomycosis is caused by dematiaceous fungi, distinguished by melanin in their cell walls [1], which enhances virulence by scavenging free radicals from phagocytic cells. These fungi are commonly found in soil, decaying vegetation, and rotten wood. Primary etiological agents include *Alternaria*, *Phialophora*, and *Exophiala* [2]. They also occur following traumatic inoculation. Clinical presentations range from solitary cutaneous nodules and verrucous plaques to deep subcutaneous abscesses [3]. Histopathology and culture are essential for diagnosis.

Immunocompromised individuals, such as those with HIV/AIDS, organ transplant recipients, patients with hematological malignancies, and those undergoing chemotherapy, are particularly susceptible to infections like phaeohyphomycosis due to several factors. These include diminished immune cell activity, particularly T cells, which are critical in defending against fungal pathogens. Additionally, the use of immunosuppressive therapies further impairs immune function, creating a favorable environment for fungal invasion. The diverse clinical manifestations of such infections present a diagnostic challenge in these patients. Furthermore, surgical procedures and the use of indwelling medical devices may facilitate fungal introduction, especially in individuals with already compromised immune defenses.

This case series presents three cases of localized cutaneous phaeohyphomycosis in renal transplant recipients, each with distinct clinical manifestations.

## Case presentation

Case 1

**Figure 1:**
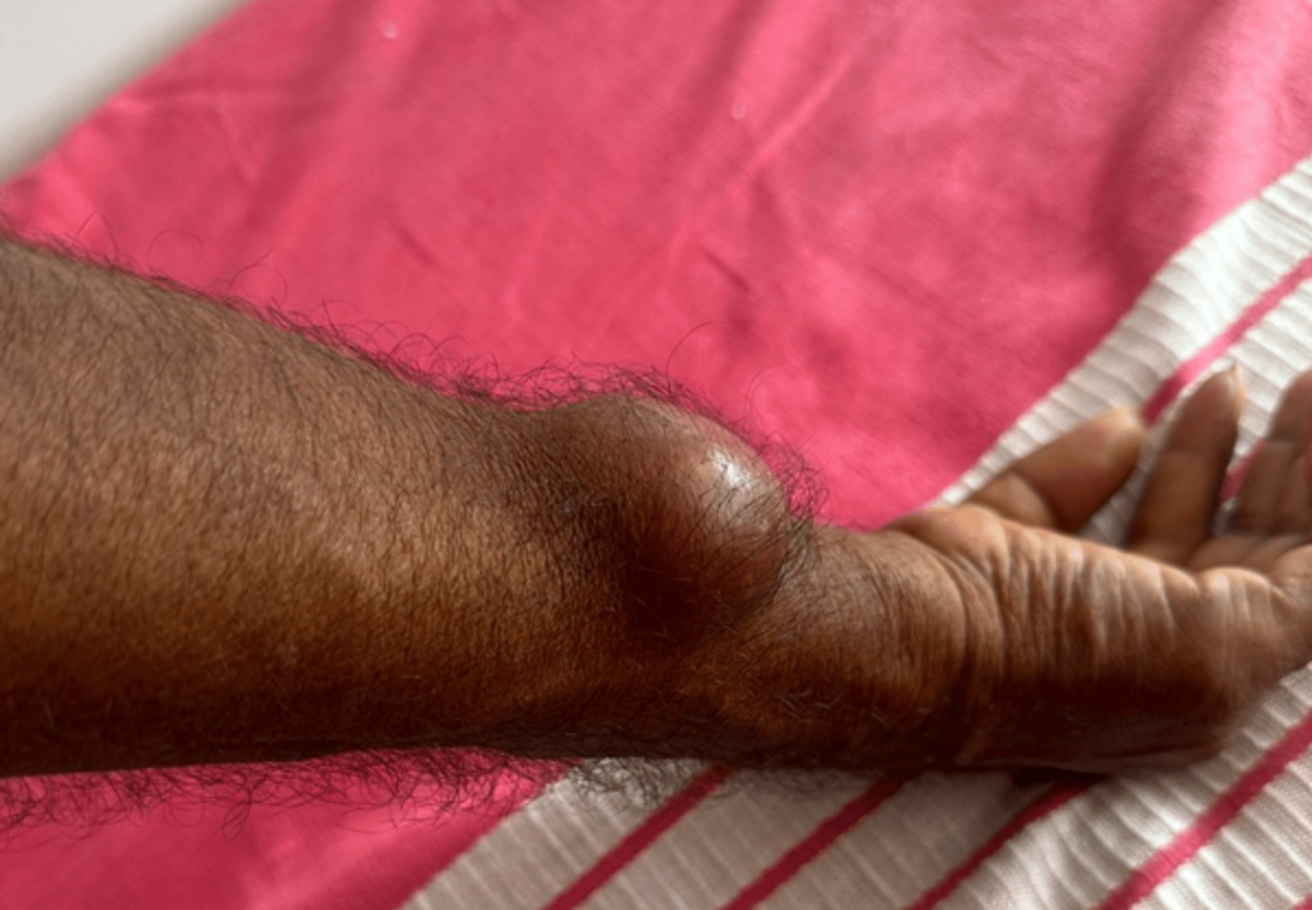
A solitary painless cyst on the right forearm measuring 6 x 4 cm.

A 46-year-old male farmer presented with a painless, single cyst on the right forearm, measuring 6 x 4 cm, persisting for eight months (Figure 1).

He had a history of diabetes mellitus and hypertension, with chronic kidney disease that progressed to end-stage renal failure. Six months post renal transplant, while on tacrolimus and mycophenolate mofetil (1 gm/day), he developed skin lesions. Laboratory tests revealed serum creatinine of 5.9 mg/dL and blood urea nitrogen of 60 mg/dL.

Case 2

**Figure 2:**
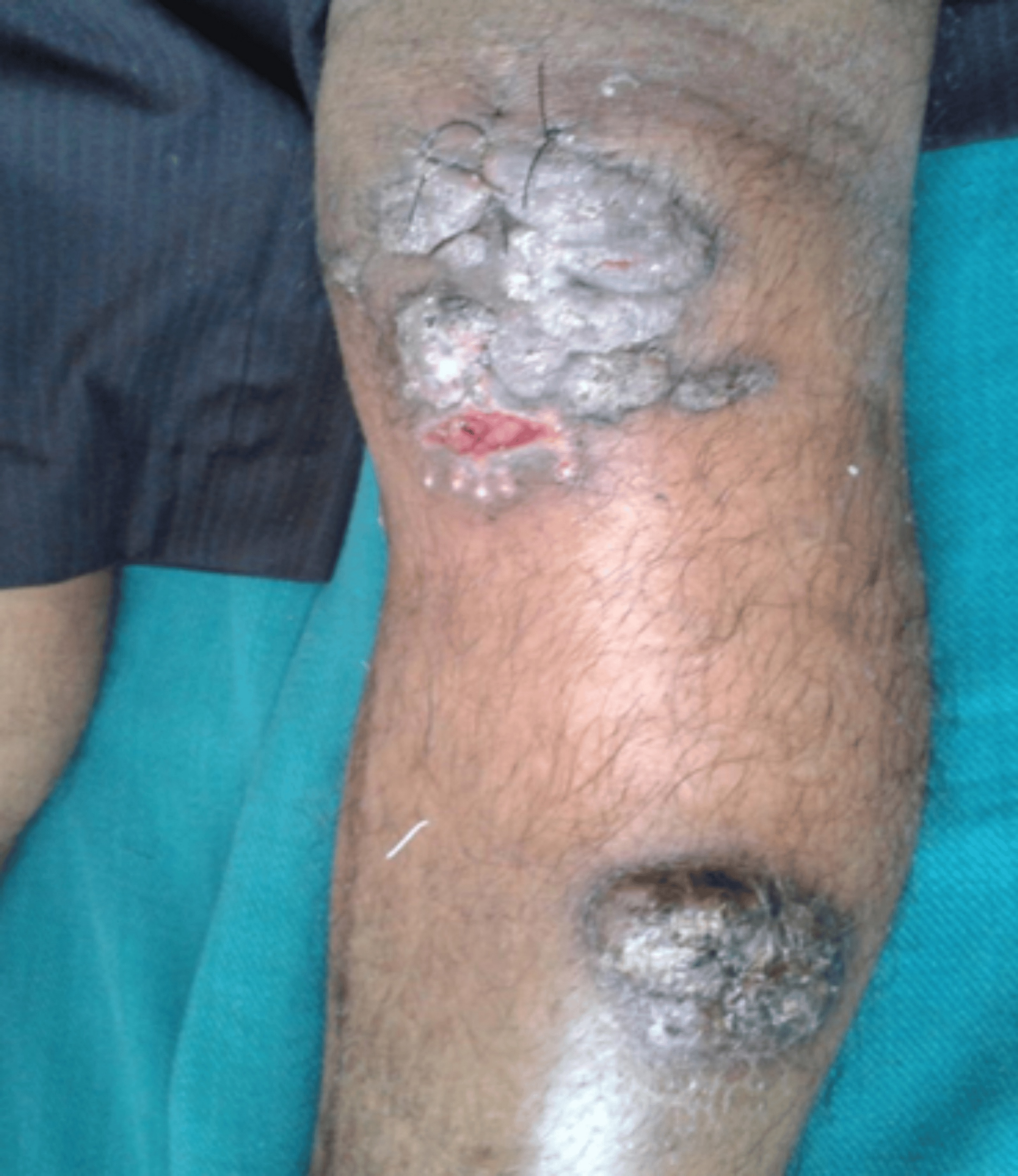
Multiple painless verrucous plaques, measuring 6 x 5 cm to 8 x 6 cm, on the left leg.

A 40-year-old male gardener presented with multiple, painless verrucous plaques following trauma on the left leg, measuring 6 x 5 cm to 8 x 6 cm, persisting for five months (Figure 2).

He had chronic glomerulonephritis, which progressed to end-stage renal failure, requiring dialysis for eight months before undergoing renal transplantation. Skin lesions appeared nine months post transplant. The patient was on mycophenolate mofetil (2 g/day). Laboratory tests showed a blood urea nitrogen of 84 mg/dL and serum creatinine of 3 mg/dL.

Case 3

**Figure 3:**
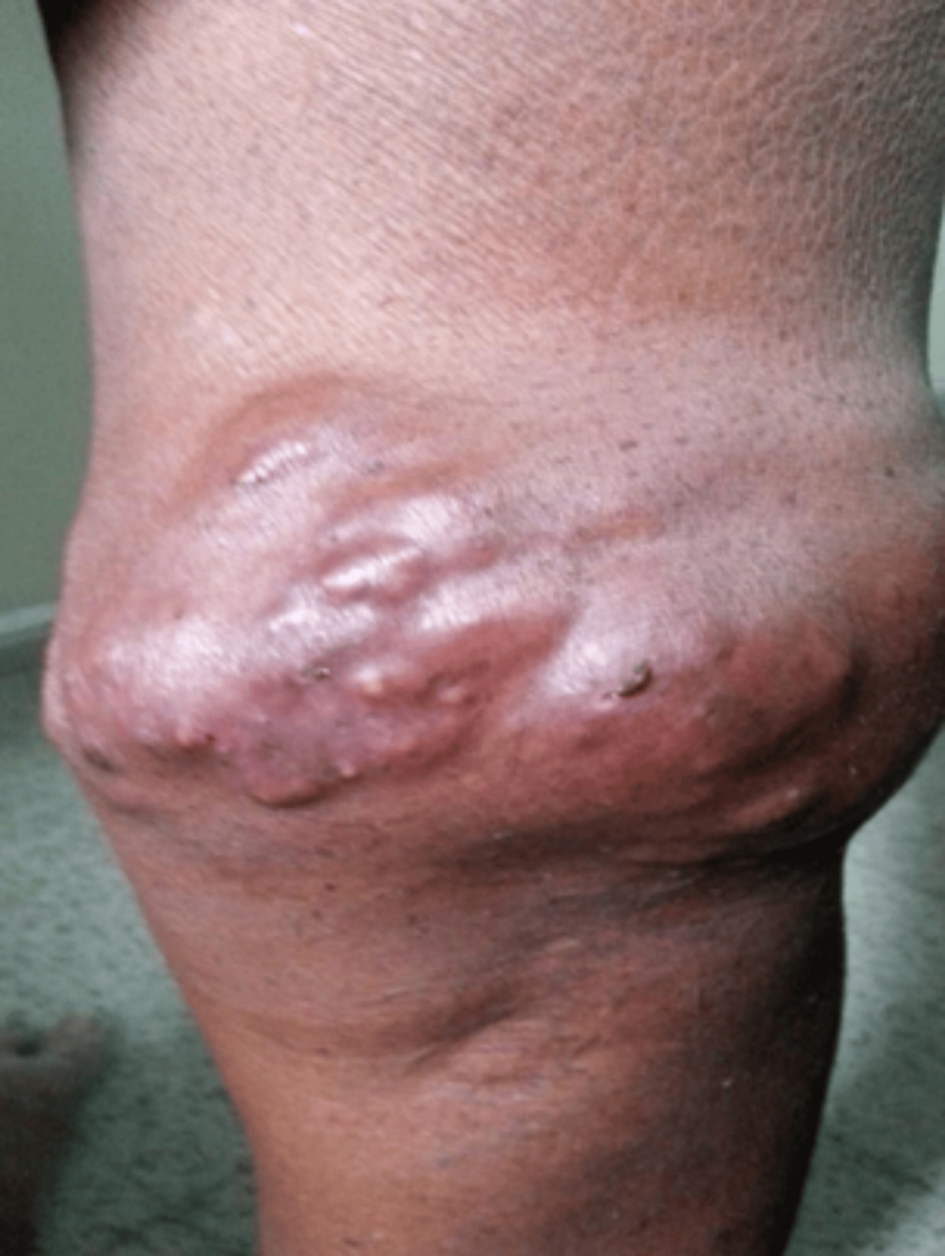
Painful bursitis in the right popliteal fossa.

A 50-year-old male shopkeeper presented with painful bursitis in the right popliteal fossa for three months (Figure 3).

He had a history of hypertension and poorly controlled diabetes, which led to end-stage kidney disease, necessitating renal transplantation. Post transplant, he developed a lesion in the right popliteal fossa. Blood urea nitrogen was 72 mg/dL, and serum creatinine was 7.8 mg/dL.

**Figure 4:**
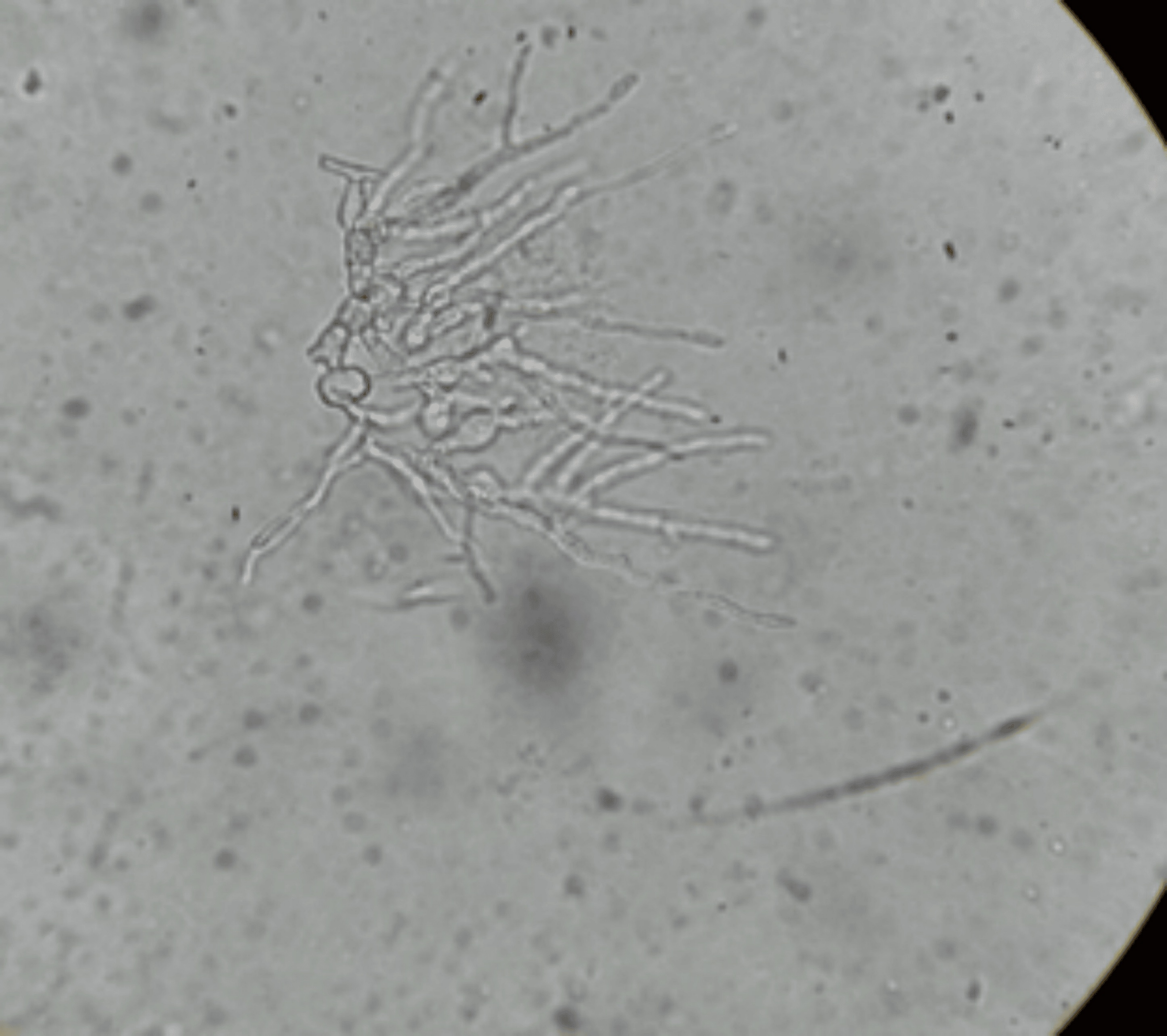
Potassium hydroxide (KOH) mount showing pigmented, septate hyphae with fusiform swellings (case 1).

Potassium hydroxide (KOH) mount test was performed for all three cases. The KOH mount from case 1 revealed pigmented, hyaline, septate, moniliform hyphae (Figure 4).

**Figure 5:**
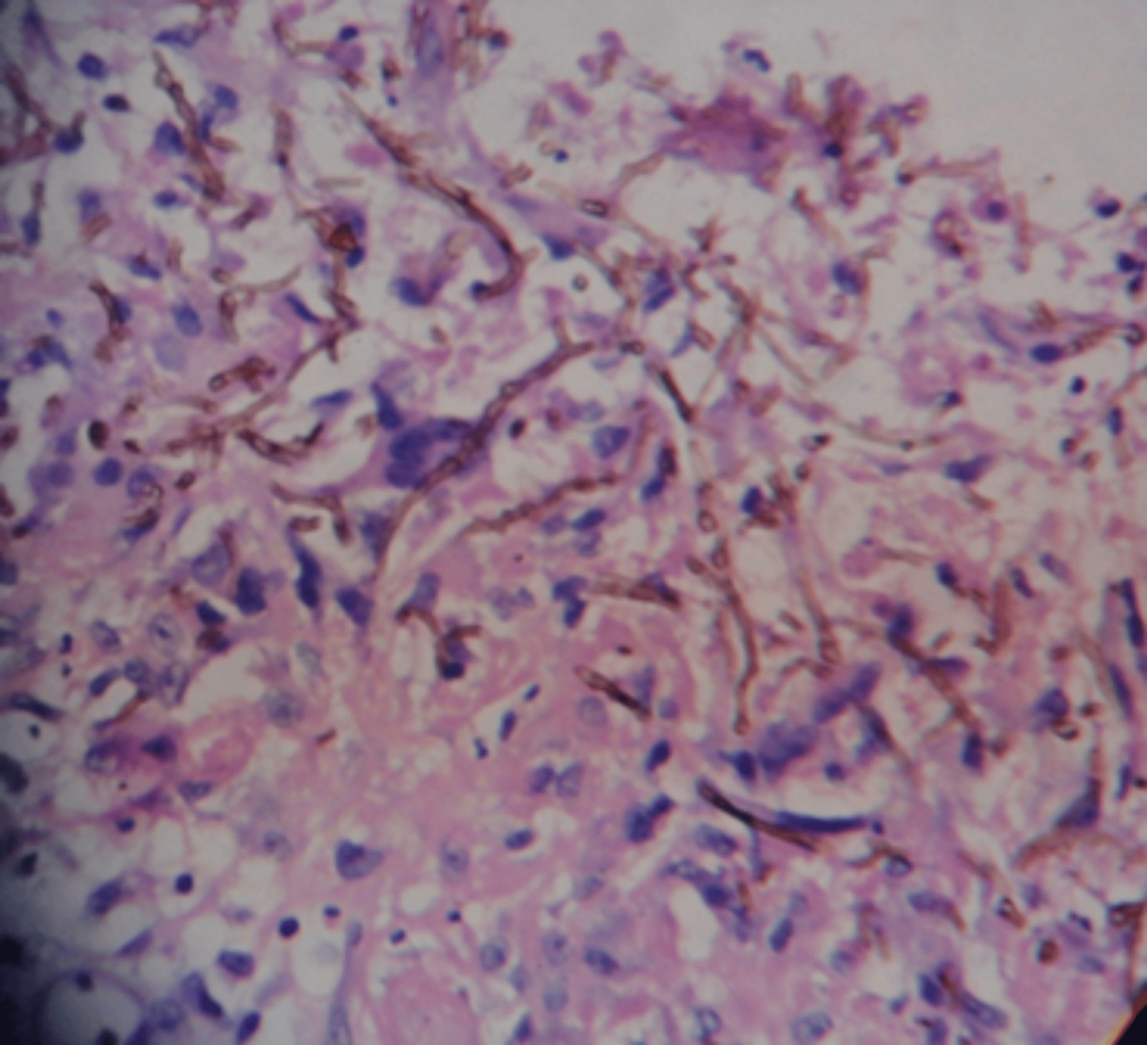
Histopathology (hematoxylin and eosin staining, 40x magnification) revealing brown pigmented hyphae (case 2).

**Figure 6:**
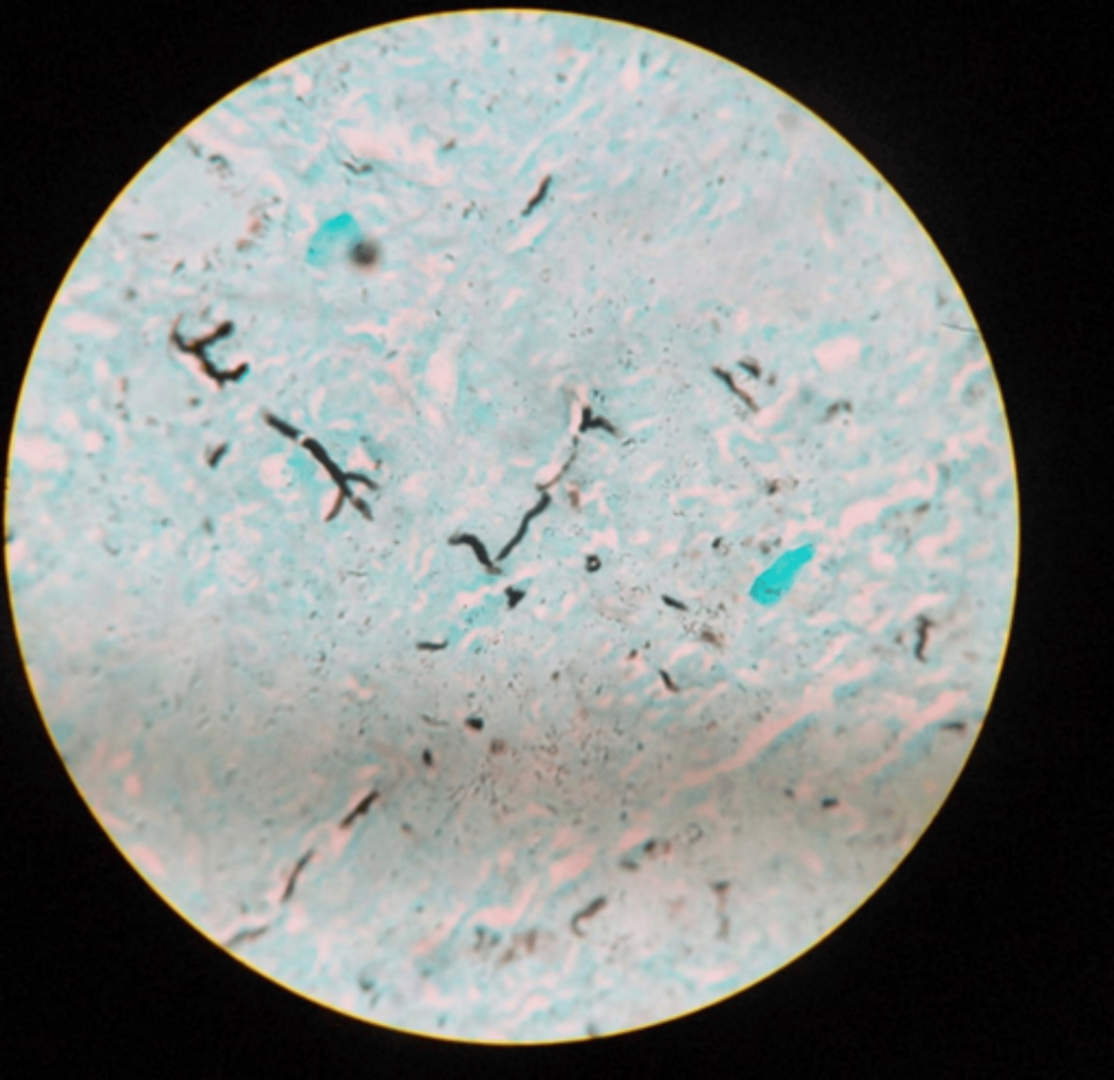
Gomori methenamine silver stain highlighting pigmented hyphae (case 2).

Histopathological examination of tissue biopsy from case 2 showed brown pigmented hyphae, and Gomori methenamine silver stain confirmed the presence of pigmented, septate hyphae (Figures 5, 6).

**Figure 7:**
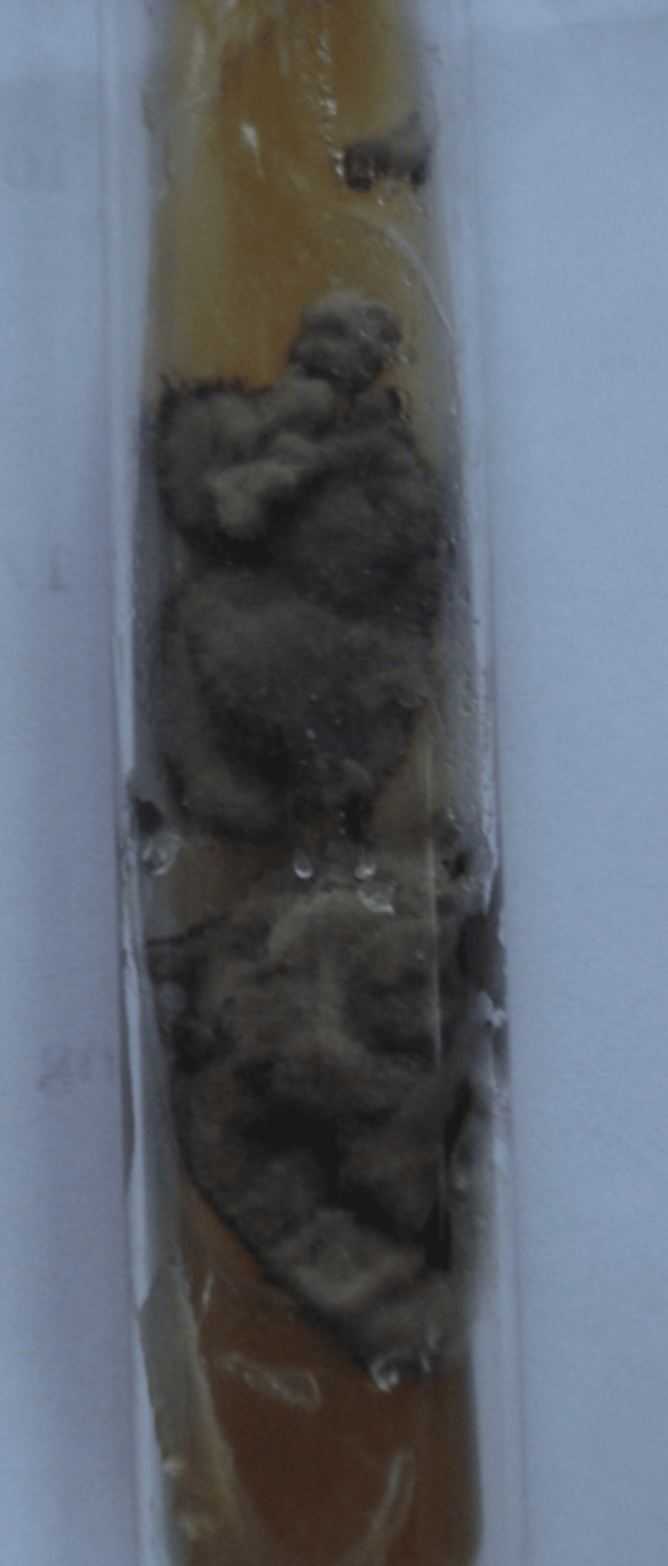
Grey colonies of Phialophora verrucosa on Sabouraud dextrose agar (case 3).

Culture on Sabouraud dextrose agar in case 3 identified *Phialophora verrucosa *(Figure 7).

None of the cases exhibited any systemic symptoms. The differential diagnoses for case 1 included ganglion cyst, lipoma, and epidermal inclusion cyst. For case 2, the differential diagnoses included verrucous tuberculosis, chromoblastomycosis, and sporotrichosis. In case 3, the differential diagnoses considered were ganglion cyst, abscess, and meniscal cyst. Treatment varied based on presentation; case 1: oral itraconazole (200 mg twice daily for three months) with surgical excision; case 2: oral voriconazole (200 mg twice daily for four months) with surgical excision; case 3: surgical excision followed by oral itraconazole (100 mg twice daily for six months).

## Discussion

The term phaeohyphomycosis was introduced by Ajello in 1974 to describe infections caused by melanized fungi [4]. These fungi exist in tissues as pigmented yeasts, pseudohyphae, or hyphal forms; they can infect both immunocompetent and immunocompromised individuals. They are predominantly found in tropical and subtropical regions, with *Exophiala jeanselmei*, *Bipolaris sp.*, and *Phialophora verrucosa* being the most common species [5]. *Cladophialophora* species are primarily associated with cerebral phaeohyphomycosis. The infection can be localized (cutaneous or subcutaneous cysts) or disseminated.

Disseminated infection occurs predominantly in immunocompromised individuals, including those with malignancies and solid organ transplants (particularly renal transplant recipients) [6]. The most common extracutaneous sites include the paranasal sinuses, lungs, and central nervous system. The clinical manifestations of phaeohyphomycosis are diverse and can include cutaneous, subcutaneous, and extracutaneous (systemic) involvement. Cutaneous and subcutaneous manifestations are the most common presentations and often occur following traumatic inoculation. The cutaneous manifestations can include papules, pustules, verrucous plaques, nodules, ulcers, or cystic swelling. Common sites of involvement include the hands, feet, forearms, and face. Subcutaneous phaeohyphomycosis typically presents as a firm, painless nodule or cyst deep in the dermis or subcutaneous tissue, which may progress to form abscesses or sinus tracts.

Extracutaneous phaeohyphomycosis is often observed in immunocompromised patients and most commonly affects the sinopulmonary and central nervous systems. Pulmonary manifestations can include sinusitis, pneumonia, or lung abscesses. Central nervous system involvement occurs via hematogenous spread or direct invasion, with brain abscesses being the typical presentation. *Cladophialophora bantiana *is a common cause of neural involvement in phaeohyphomycosis. Central nervous system involvement is fatal if not diagnosed early.

Histopathology typically reveals granulomas with pigmented hyphae and pseudohyphae [7]. These fungi grow on Sabouraud dextrose agar, cornmeal agar, and malt extract agar, forming olive-to-dark brown colonies. Differential diagnoses include lipomas, fibromas, epidermal cysts [8], and other infectious conditions such as mycetoma, cutaneous tuberculosis, chromoblastomycosis, leishmaniasis, and sporotrichosis.

There are no standardized treatment guidelines for phaeohyphomycosis. Surgical excision can be done for localized cysts, while disseminated infections require antifungal therapy, including itraconazole, voriconazole, posaconazole, or amphotericin B, frequently combined with surgical debridement. The prognosis is favorable for localized infections, but disseminated cases have a high mortality rate exceeding 70% [9].

Prasad et al. [10] emphasized the importance of early diagnosis, combining antifungal therapy and surgical excision for effective treatment. Their findings showed varying outcomes, with some cases achieving complete resolution, while others had partial regression or mortality due to disseminated infection. Similarly, Singla et al. [11] reported cases where surgical debridement led to complete resolution.

Thus, the present case series reinforces the importance of considering phaeohyphomycosis as a differential diagnosis in transplant recipients presenting with persistent skin or subcutaneous lesions. Furthermore, they emphasize the necessity for heightened suspicion of fungal infections in immunocompromised individuals, where early diagnosis using KOH preparation may prove crucial for patient survival. A combined approach of medical and surgical interventions yields the most favorable outcomes. Notably, the absence of systemic symptoms in these cases suggests a localized infection. Additionally, imaging and thorough evaluation are critical in cases of systemic phaeohyphomycosis, particularly disseminated infections, to aid in lesion characterization, guide biopsies, and facilitate ongoing monitoring.

## Conclusions

Phaeohyphomycosis presents a diagnostic challenge due to its diverse clinical manifestations, frequently mimicking other conditions. A high index of suspicion for deep fungal infections is essential, as even a simple KOH examination can aid in diagnosis. Early detection reduces mortality, limits disease progression, and improves patient outcomes.
